# 9-(3-Bromo-5-chloro-2-hy­droxy­phen­yl)-10-(2-hy­droxy­eth­yl)-3,6-diphenyl-3,4,9,10-tetra­hydro­acridine-1,8(2*H*,5*H*)-dione

**DOI:** 10.1107/S1600536814010460

**Published:** 2014-05-17

**Authors:** Mehmet Akkurt, Shaaban K. Mohamed, Antar A. Abdelhamid, Abdel-Aal M. Gaber, Mustafa R. Albayati

**Affiliations:** a Department of Physics, Faculty of Sciences, Erciyes University, 38039 Kayseri, Turkey; b Chemistry and Environmental Division, Manchester Metropolitan University, Manchester M1 5GD, England; c Chemistry Department, Faculty of Science, Minia University, 61519 El-Minia, Egypt; d Chemistry Department, Faculty of Science, Sohag University, 82524 Sohag, Egypt; e Department of Chemistry, Faculty of Science, Assiut University, 71516 Assiut, Egypt; f Kirkuk University, College of Science, Department of Chemistry, Kirkuk, Iraq

## Abstract

In the title compound, C_33_H_27_BrClNO_4_, the di­hydro­pyridine ring adopts a flattened boat conformation. The mol­ecular conformation is stabilized by an intra­molecular O—H⋯O hydrogen bond, with an *S*(8) ring motif. In the crystal, O—H⋯O, C—H⋯O and C—H⋯Cl hydrogen bonds, and C—H⋯π inter­actions link the mol­ecules, forming a three-dimensional network. In the acridinedione ring system, the two ring C atoms at the 2- and 3-positions, and the C atom at the 6-position and the atoms of the phenyl ring attached to the C atom at the 6-position are disordered over two sets of sites with occupancy ratios of 0.783 (5):0.217 (5) and 0.526 (18):0.474 (18), respectively.

## Related literature   

For different industrial applications of acridine-1,8-diones, see: Murugan *et al.* (1998 ▶); Srividya *et al.* (1996 ▶, 1998 ▶). For various pharmaceutical properties of acridine-containing compounds, see: Girault *et al.* (2000 ▶); Sánchez *et al.* (2006 ▶); Astelbauer *et al.* (2011 ▶); Yang *et al.* (2006 ▶); Shaikh *et al.* (2010 ▶); Gunduz *et al.* (2009 ▶). For hydrogen-bond motifs, see: Bernstein *et al.* (1995 ▶). For related structures, see: Mohamed *et al.* (2013 ▶); Sughanya & Sureshbabu (2012 ▶); Yogavel *et al.* (2005 ▶).
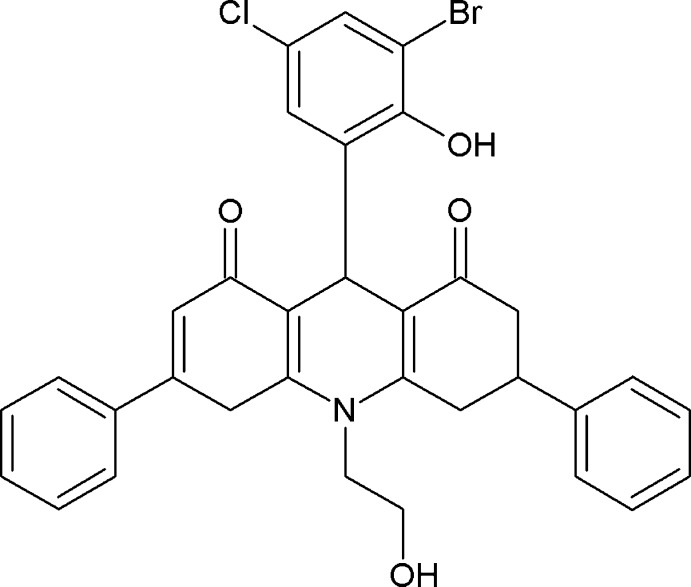


## Experimental   

### 

#### Crystal data  

C_33_H_27_BrClNO_4_
*M* *_r_* = 616.91
Monoclinic, 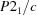

*a* = 14.7307 (3) Å
*b* = 15.4874 (3) Å
*c* = 13.6541 (3) Å
β = 107.110 (2)°
*V* = 2977.18 (11) Å^3^
*Z* = 4
Mo *K*α radiation
μ = 1.51 mm^−1^
*T* = 293 K
0.20 × 0.09 × 0.09 mm

#### Data collection  

Oxford Diffraction Xcalibur CCD diffractometer
Absorption correction: analytical (*CrysAlis RED*; Oxford Diffraction, 2003 ▶) *T*_min_ = 0.631, *T*_max_ = 0.791
45181 measured reflections
9225 independent reflections
4420 reflections with *I* > 2σ(*I*)
*R*_int_ = 0.037

#### Refinement  

*R*[*F* ^2^ > 2σ(*F* ^2^)] = 0.050
*wR*(*F* ^2^) = 0.147
*S* = 0.92
9225 reflections
347 parameters
107 restraints
H atoms treated by a mixture of independent and constrained refinement
Δρ_max_ = 0.60 e Å^−3^
Δρ_min_ = −0.40 e Å^−3^

### 

Data collection: *CrysAlis CCD* (Oxford Diffraction, 2003 ▶); cell refinement: *CrysAlis CCD*; data reduction: *CrysAlis RED* (Oxford Diffraction, 2003 ▶); program(s) used to solve structure: *SHELXS97* (Sheldrick, 2008 ▶); program(s) used to refine structure: *SHELXL97* (Sheldrick, 2008 ▶); molecular graphics: *ORTEP-3 for Windows* (Farrugia, 2012 ▶); software used to prepare material for publication: *WinGX* (Farrugia, 2012 ▶) and *PLATON* (Spek, 2009 ▶).

## Supplementary Material

Crystal structure: contains datablock(s) global, I. DOI: 10.1107/S1600536814010460/hg5395sup1.cif

Structure factors: contains datablock(s) I. DOI: 10.1107/S1600536814010460/hg5395Isup2.hkl

Supporting information file. DOI: 10.1107/S1600536814010460/hg5395Isup3.cml

CCDC reference: 1001670

Additional supporting information:  crystallographic information; 3D view; checkCIF report

## Figures and Tables

**Table 1:** Hydrogen-bond geometry (Å, °) *Cg*6, *Cg*7 and *Cg*9 are the centroids of the C28B_B–C33B_B, C14–C19 and C28A_A–C33A_A phenyl rings, respectively.

*D*—H⋯*A*	*D*—H	H⋯*A*	*D*⋯*A*	*D*—H⋯*A*
O2—H2*O*⋯O1^i^	0.87 (2)	1.93 (2)	2.782 (3)	167 (5)
O4—H4*O*⋯O1	0.83 (3)	1.84 (3)	2.632 (2)	161 (3)
C10—H10*A*⋯O3^ii^	0.97 (2)	2.54 (2)	3.211 (3)	126 (2)
C31*B*_*b*—H31*B*_*b*⋯Cl1^iii^	0.93	2.76	3.530 (7)	141
C26—H26*B*⋯O3^ii^	0.97	2.57	3.537 (3)	173
C16—H16⋯*Cg*6^iv^	0.93	2.89	3.713 (4)	149
C16—H16⋯*Cg*9^iv^	0.93	2.86	3.718 (4)	154
C27—H27*B*⋯*Cg*7^v^	0.97	2.71	3.574 (3)	149

Symmetry codes: (i) 
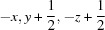
; (ii) 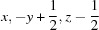; (iii) 
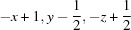
; (iv) 
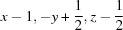
; (v) 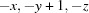.

**Fig. 1.**
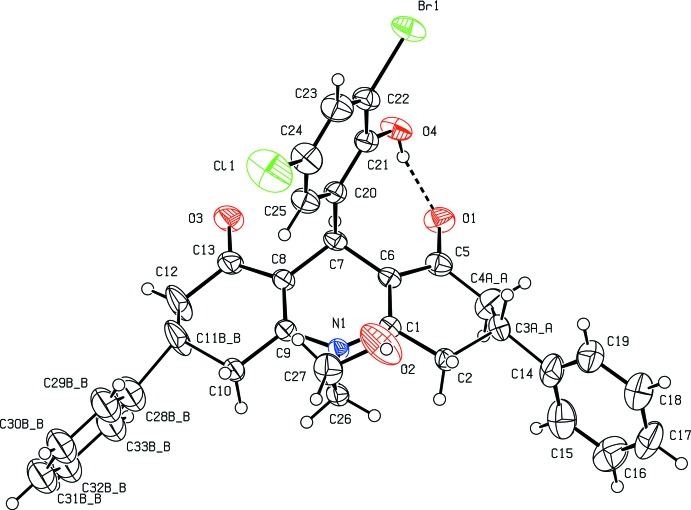
View of the title compound with 30% probability displacement ellipsoids. Only the major components of the disorders are shown.

**Fig. 2.**
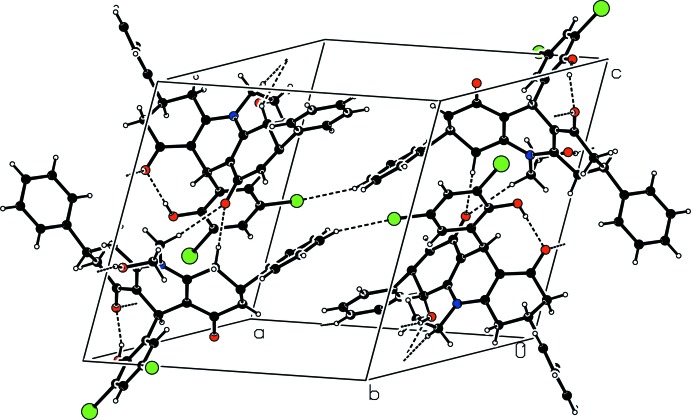
Perspective view of the hydrogen bonding and packing of the title compound. Only the major components of the disorders are shown.

## supplementary crystallographic information

### 1. Comment

Substituted acridinediones are an interested class of heterocyclic compounds due to their wide industrial and medicinal applications. Acridinones considered to be one of earlist antibiotics. Acridinone scakffold compounds exhibit various bioactivities such as, anti-malerial (Girault *et al.*, 2000), anti-tumor (Sánchez *et al.*, 2006), anti-leishmanial activities (Astelbauer *et al.*, 2011), DNA-binding and DNA photo-damaging ability (Yang *et al.*, 2006), antimicrobial activity (Shaikh *et al.*, 2010) and potassium channel blockers (Gunduz *et al.*, 2009). Certain acridine-1,8-diones showed fluorescence activities (Murugan *et al.*, 1998) and a few acridinedione derivatives also show photophysical (Srividya *et al.*, 1998) and electrochemical properties (Srividya *et al.*, 1996). Thus, the accurate description of crystal structures of substituted acridinediones are expected to provide useful information.

In the title compound (I, Fig. 1), the dihydropyridine ring (N1/C1/C6–C9) is nearly planar with a maximum deviation of 0.225 (2) Å for C7. The C14–C19 phenyl and C20–C25 benzene rings form dihedral angles of 73.40 (10) and 83.32 (11)°, respectively, with the dihydropyridine mean plane. The dihedral angle between the C28A–C33A and C28B and C33B disordered phenyl rings is 16.3 (4) °.

In (I), all bond lengths and angles are within normal ranges and and comparable with those in related similar compounds (Mohamed *et al.*, 2013; Sughanya & Sureshbabu, 2012; Yogavel *et al.*, 2005). The ethanol group attached to the 1,4-dihydropyridine ring has a N1—C26—C27—O2 torsion angle of -76.8 (3)°.

The molecular conformation of (I) is stabilized by an intramolecular O—H···O hydrogen bond (Table 1), which forms a pseudo-eight-membered ring with graph set S(8) (Bernstein *et al.*, 1995).

In the crystal, molecules are linked by O—H···O, C—H···O and C—H···Cl hydrogen bonds, forming three dimensional network (Table 1, Fig. 2). Furthermore, C—H···π interactions (Table 1) contribute to the stabilization of the molecular packing.

### 2. Experimental

A mixture of 1 mmol (235 mg) of 3-bromo-5-chloro-2-hydroxybenzaldehyde, 2 mmol (372 mg) of 5-phenylcyclohexane-1,3-dione and 1 mmol (61 mg) of 2-aminoethanol in 30 ml e thanol was refluxed for 2 h at 350 K. The reaction mixture was cooled at ambient temperature and the precipitated product was filtered off, washed with cold ethanol and recrystallized from ethanol. Suitable crystals for X-ray diffractions were obtained by slow evaporation method of an ethanolic solution of (I) at room temperature over two days.

### 3. Refinement

The hydroxyl H atoms were found from a difference Fourier map [O2—H2O = 0.873 (19) Å and O4—H4O = 0.826 (17) Å]. Their coordinates were freely refined and *U*_iso_(H) were set to 1.5*U*_eq_(O). The H atoms attached to C2 and C12 were located in a difference map and refined freely. The other H-atoms were placed in calculated positions and refined by using a riding model with C—H = 0.93 – 0.98 Å [*U*_iso_(H) = 1.2 *U*_eq_(C)].

In the 3,4,9,10-tetrahydroacridine-1,8(2*H*,5H)-dione ring system, the two ring C atoms (C3 and C4) at the 2 and 3-poisitions are disordered over two positions with the site occupancy factors of 0.783 (5) and 0.217 (5). For the C4A and C4B atoms of disorder, the EXYZ instruction was used in the refinement.

The C atom (C11) at the 6-positions of the mentioned ring system and the atoms of the phenyl ring (C28–C33) attached to the C11 atom are disordered over two positions; the site occupancy factors are 0.526 (18) and 0.474 (18).

The atoms of disorder were set to equal each other by an EADP. The disordered phenyl ring (C28A/B–C33A/B) was constrained to a rigid hexagon with the AFIX 66 instruction, and the SIMU and DELU instructions were used in the refinement procedure.

### Crystal data

**Table:**

C_33_H_27_BrClNO_4_	*F*(000) = 1264
*M**_r_* = 616.91	*D*_x_ = 1.376 Mg m^−^^3^
Monoclinic, *P*2_1_/*c*	Mo *K*α radiation, λ = 0.71073 Å
*a* = 14.7307 (3) Å	Cell parameters from 729 reflections
*b* = 15.4874 (3) Å	θ = 4–45°
*c* = 13.6541 (3) Å	µ = 1.51 mm^−^^1^
β = 107.110 (2)°	*T* = 293 K
*V* = 2977.18 (11) Å^3^	Prism, colourless
*Z* = 4	0.20 × 0.09 × 0.09 mm

### Data collection

**Table:**

Oxford Diffraction Xcalibur CCD diffractometer	9225 independent reflections
Radiation source: fine-focus sealed tube	4420 reflections with *I* > 2σ(*I*)
Graphite monochromator	*R*_int_ = 0.037
ω scans	θ_max_ = 31.5°, θ_min_ = 3.8°
Absorption correction: analytical (*CrysAlis RED*; Oxford Diffraction, 2003)	*h* = −21→21
*T*_min_ = 0.631, *T*_max_ = 0.791	*k* = −20→22
45181 measured reflections	*l* = −19→19

### Refinement

**Table:**

Refinement on *F*^2^	107 restraints
Least-squares matrix: full	Hydrogen site location: mixed
*R*[*F*^2^ > 2σ(*F*^2^)] = 0.050	H atoms treated by a mixture of independent and constrained refinement
*wR*(*F*^2^) = 0.147	*w* = 1/[σ^2^(*F*_o_^2^) + (0.0793*P*)^2^] where *P* = (*F*_o_^2^ + 2*F*_c_^2^)/3
*S* = 0.92	(Δ/σ)_max_ < 0.001
9225 reflections	Δρ_max_ = 0.60 e Å^−^^3^
347 parameters	Δρ_min_ = −0.40 e Å^−^^3^

### Special details

**Table:**

Geometry. All e.s.d.'s (except the e.s.d. in the dihedral angle between two l.s. planes) are estimated using the full covariance matrix. The cell e.s.d.'s are taken into account individually in the estimation of e.s.d.'s in distances, angles and torsion angles; correlations between e.s.d.'s in cell parameters are only used when they are defined by crystal symmetry. An approximate (isotropic) treatment of cell e.s.d.'s is used for estimating e.s.d.'s involving l.s. planes.

### Fractional atomic coordinates and isotropic or equivalent isotropic displacement parameters (Å^2^)

**Table:**

	*x*	*y*	*z*	*U*_iso_*/*U*_eq_	Occ. (<1)
C1	0.01625 (15)	0.34409 (13)	0.18821 (16)	0.0299 (5)	
C2	−0.07136 (16)	0.37567 (16)	0.10912 (18)	0.0343 (5)	
H2A	−0.0682 (14)	0.3633 (13)	0.0439 (14)	0.053 (7)*	
H2B	−0.0689 (17)	0.4362 (10)	0.0999 (18)	0.041 (7)*	
C3A_a	−0.1607 (2)	0.3643 (2)	0.1427 (2)	0.0379 (8)	0.783 (5)
H3A_a	−0.1603	0.4087	0.1939	0.045*	0.783 (5)
C4A_a	−0.1630 (2)	0.2785 (2)	0.1905 (2)	0.0665 (9)	0.783 (5)
H4A1_a	−0.1655	0.2333	0.1406	0.080*	0.783 (5)
H4A2_a	−0.2193	0.2740	0.2132	0.080*	0.783 (5)
C3B_b	−0.1547 (7)	0.3181 (8)	0.1084 (9)	0.0379 (8)	0.217 (5)
H3B_b	−0.1537	0.2718	0.0598	0.045*	0.217 (5)
C4B_b	−0.1630 (2)	0.2785 (2)	0.1905 (2)	0.0665 (9)	0.217 (5)
H4B1_b	−0.1889	0.2215	0.1699	0.080*	0.217 (5)
H4B2_b	−0.2096	0.3098	0.2141	0.080*	0.217 (5)
C5	−0.07640 (18)	0.26775 (16)	0.27956 (19)	0.0430 (6)	
C6	0.01216 (15)	0.30127 (13)	0.27458 (16)	0.0316 (5)	
C7	0.10177 (15)	0.28391 (13)	0.36056 (16)	0.0316 (5)	
H7	0.0922	0.2310	0.3957	0.038*	
C8	0.18040 (16)	0.26804 (14)	0.31428 (16)	0.0338 (5)	
C9	0.18093 (15)	0.30655 (13)	0.22528 (16)	0.0321 (5)	
C10	0.26051 (17)	0.29515 (18)	0.17851 (19)	0.0423 (6)	
H10A	0.2387 (19)	0.2681 (16)	0.1118 (15)	0.061 (8)*	
H10B	0.288 (2)	0.3509 (15)	0.174 (3)	0.109 (13)*	
C11A_a	0.3291 (9)	0.2237 (11)	0.2214 (11)	0.1126 (18)	0.474 (18)
C11B_b	0.3472 (8)	0.2539 (10)	0.2571 (10)	0.1126 (18)	0.526 (18)
C12	0.3348 (3)	0.1940 (3)	0.3190 (3)	0.1126 (18)	
H12	0.3804 (19)	0.1599 (18)	0.357 (2)	0.092 (11)*	
C13	0.25648 (19)	0.20872 (17)	0.36687 (19)	0.0486 (6)	
C14	−0.24811 (12)	0.38033 (14)	0.04824 (15)	0.0603 (8)	
C15	−0.28188 (15)	0.32740 (11)	−0.03696 (18)	0.0739 (9)	
H15	−0.2522	0.2750	−0.0404	0.089*	
C16	−0.35997 (16)	0.35285 (15)	−0.11694 (15)	0.0804 (10)	
H16	−0.3826	0.3174	−0.1739	0.096*	
C17	−0.40429 (13)	0.43123 (16)	−0.11173 (15)	0.0801 (11)	
H17	−0.4565	0.4483	−0.1653	0.096*	
C18	−0.37052 (15)	0.48416 (13)	−0.02654 (18)	0.0781 (10)	
H18	−0.4002	0.5366	−0.0231	0.094*	
C19	−0.29243 (14)	0.45871 (14)	0.05344 (14)	0.0694 (9)	
H19	−0.2698	0.4941	0.1104	0.083*	
C20	0.12371 (16)	0.35813 (14)	0.43954 (17)	0.0347 (5)	
C21	0.07775 (17)	0.36271 (14)	0.51549 (17)	0.0371 (5)	
C22	0.09776 (19)	0.43067 (15)	0.58480 (19)	0.0449 (6)	
C23	0.1604 (2)	0.49502 (18)	0.5784 (2)	0.0600 (8)	
H23	0.1723	0.5410	0.6243	0.072*	
C24	0.2049 (2)	0.49020 (17)	0.5035 (2)	0.0587 (7)	
C25	0.18721 (19)	0.42243 (16)	0.4347 (2)	0.0476 (6)	
H25	0.2184	0.4201	0.3847	0.057*	
C26	0.11376 (17)	0.41559 (15)	0.09010 (18)	0.0393 (6)	
H26A	0.0526	0.4237	0.0392	0.047*	
H26B	0.1567	0.3903	0.0562	0.047*	
C27	0.1515 (2)	0.50082 (18)	0.1339 (2)	0.0592 (8)	
H27A	0.2028	0.4929	0.1968	0.071*	
H27B	0.1759	0.5325	0.0857	0.071*	
C28A_a	0.4156 (5)	0.2360 (6)	0.1926 (6)	0.103 (2)	0.474 (18)
C29A_a	0.4818 (5)	0.3021 (6)	0.2049 (9)	0.103 (2)	0.474 (18)
H29A_a	0.4817	0.3476	0.2494	0.124*	0.474 (18)
C30A_a	0.5482 (5)	0.3004 (6)	0.1507 (9)	0.103 (2)	0.474 (18)
H30A_a	0.5925	0.3446	0.1589	0.124*	0.474 (18)
C31A_a	0.5484 (4)	0.2325 (8)	0.0842 (6)	0.103 (2)	0.474 (18)
H31A_a	0.5928	0.2313	0.0479	0.124*	0.474 (18)
C32A_a	0.4822 (5)	0.1664 (9)	0.0718 (6)	0.103 (2)	0.474 (18)
H32A_a	0.4823	0.1209	0.0273	0.124*	0.474 (18)
C33A_a	0.4157 (4)	0.1681 (7)	0.1261 (6)	0.103 (2)	0.474 (18)
H33A_a	0.3714	0.1239	0.1178	0.124*	0.474 (18)
C28B_b	0.4134 (4)	0.2230 (5)	0.1810 (5)	0.0843 (16)	0.526 (18)
C29B_b	0.4696 (5)	0.2920 (4)	0.1691 (7)	0.0843 (16)	0.526 (18)
H29B_b	0.4623	0.3458	0.1962	0.101*	0.526 (18)
C30B_b	0.5366 (5)	0.2807 (5)	0.1168 (7)	0.0843 (16)	0.526 (18)
H30B_b	0.5742	0.3269	0.1089	0.101*	0.526 (18)
C31B_b	0.5475 (4)	0.2003 (6)	0.0764 (4)	0.0843 (16)	0.526 (18)
H31B_b	0.5924	0.1927	0.0414	0.101*	0.526 (18)
C32B_b	0.4914 (5)	0.1313 (6)	0.0882 (6)	0.0843 (16)	0.526 (18)
H32B_b	0.4987	0.0775	0.0612	0.101*	0.526 (18)
C33B_b	0.4243 (5)	0.1426 (5)	0.1406 (6)	0.0843 (16)	0.526 (18)
H33B_b	0.3868	0.0964	0.1485	0.101*	0.526 (18)
N1	0.10280 (12)	0.35608 (11)	0.17027 (13)	0.0311 (4)	
O1	−0.08277 (13)	0.22579 (12)	0.35667 (13)	0.0538 (5)	
O2	0.0767 (2)	0.54656 (14)	0.1538 (2)	0.0989 (9)	
H2O	0.080 (3)	0.6015 (12)	0.141 (4)	0.148*	
O3	0.25495 (15)	0.17139 (14)	0.44472 (14)	0.0661 (6)	
O4	0.01542 (13)	0.30233 (11)	0.52701 (13)	0.0504 (5)	
H4O	−0.006 (2)	0.2696 (17)	0.4779 (19)	0.076*	
Cl1	0.28416 (9)	0.57108 (6)	0.49363 (10)	0.1102 (4)	
Br1	0.03561 (2)	0.43737 (2)	0.68766 (2)	0.06519 (14)	

### Atomic displacement parameters (Å^2^)

**Table:**

	*U*^11^	*U*^22^	*U*^33^	*U*^12^	*U*^13^	*U*^23^
C1	0.0332 (12)	0.0253 (11)	0.0332 (11)	−0.0002 (9)	0.0127 (10)	−0.0004 (9)
C2	0.0320 (12)	0.0377 (13)	0.0357 (12)	0.0023 (10)	0.0140 (10)	0.0058 (10)
C3A_a	0.0337 (15)	0.0334 (18)	0.0492 (19)	0.0019 (14)	0.0163 (14)	−0.0012 (14)
C4A_a	0.0506 (17)	0.087 (2)	0.0586 (18)	−0.0269 (16)	0.0103 (14)	0.0211 (16)
C3B_b	0.0337 (15)	0.0334 (18)	0.0492 (19)	0.0019 (14)	0.0163 (14)	−0.0012 (14)
C4B_b	0.0506 (17)	0.087 (2)	0.0586 (18)	−0.0269 (16)	0.0103 (14)	0.0211 (16)
C5	0.0484 (15)	0.0432 (14)	0.0422 (13)	−0.0068 (12)	0.0206 (12)	0.0019 (11)
C6	0.0379 (12)	0.0287 (11)	0.0322 (12)	−0.0004 (10)	0.0165 (10)	0.0009 (9)
C7	0.0409 (13)	0.0276 (11)	0.0309 (11)	0.0020 (10)	0.0177 (10)	0.0046 (9)
C8	0.0375 (13)	0.0347 (12)	0.0323 (11)	0.0046 (10)	0.0150 (10)	0.0018 (9)
C9	0.0338 (12)	0.0314 (12)	0.0333 (11)	0.0027 (9)	0.0131 (10)	0.0020 (9)
C10	0.0365 (13)	0.0557 (16)	0.0390 (14)	0.0121 (12)	0.0177 (11)	0.0113 (12)
C11A_a	0.091 (2)	0.181 (4)	0.095 (3)	0.103 (3)	0.072 (2)	0.096 (3)
C11B_b	0.091 (2)	0.181 (4)	0.095 (3)	0.103 (3)	0.072 (2)	0.096 (3)
C12	0.091 (2)	0.181 (4)	0.095 (3)	0.103 (3)	0.072 (2)	0.096 (3)
C13	0.0544 (16)	0.0581 (16)	0.0371 (13)	0.0196 (13)	0.0193 (12)	0.0136 (12)
C14	0.0332 (14)	0.076 (2)	0.0701 (19)	−0.0117 (15)	0.0125 (14)	0.0273 (17)
C15	0.0568 (19)	0.0585 (19)	0.109 (3)	−0.0041 (16)	0.028 (2)	0.016 (2)
C16	0.070 (2)	0.082 (2)	0.078 (2)	−0.032 (2)	0.0037 (19)	−0.0097 (19)
C17	0.0467 (18)	0.094 (3)	0.082 (3)	−0.0083 (18)	−0.0080 (17)	0.019 (2)
C18	0.059 (2)	0.079 (2)	0.089 (2)	0.0096 (18)	0.0099 (19)	0.008 (2)
C19	0.0482 (18)	0.088 (2)	0.068 (2)	−0.0023 (17)	0.0101 (16)	0.0021 (17)
C20	0.0379 (13)	0.0334 (12)	0.0334 (12)	0.0043 (10)	0.0114 (10)	0.0031 (9)
C21	0.0423 (13)	0.0342 (12)	0.0353 (12)	0.0043 (11)	0.0124 (11)	0.0024 (10)
C22	0.0547 (16)	0.0429 (15)	0.0407 (13)	0.0053 (12)	0.0196 (12)	−0.0043 (11)
C23	0.073 (2)	0.0482 (17)	0.0587 (18)	−0.0042 (15)	0.0200 (16)	−0.0182 (14)
C24	0.0642 (19)	0.0440 (16)	0.0725 (19)	−0.0157 (14)	0.0271 (16)	−0.0104 (14)
C25	0.0560 (16)	0.0444 (15)	0.0479 (15)	−0.0057 (12)	0.0237 (13)	−0.0008 (11)
C26	0.0393 (13)	0.0472 (14)	0.0365 (12)	0.0096 (11)	0.0189 (11)	0.0170 (10)
C27	0.0572 (18)	0.0572 (17)	0.0693 (19)	−0.0030 (14)	0.0278 (15)	0.0220 (15)
C28A_a	0.067 (3)	0.135 (4)	0.123 (3)	0.048 (3)	0.053 (3)	0.076 (3)
C29A_a	0.067 (3)	0.135 (4)	0.123 (3)	0.048 (3)	0.053 (3)	0.076 (3)
C30A_a	0.067 (3)	0.135 (4)	0.123 (3)	0.048 (3)	0.053 (3)	0.076 (3)
C31A_a	0.067 (3)	0.135 (4)	0.123 (3)	0.048 (3)	0.053 (3)	0.076 (3)
C32A_a	0.067 (3)	0.135 (4)	0.123 (3)	0.048 (3)	0.053 (3)	0.076 (3)
C33A_a	0.067 (3)	0.135 (4)	0.123 (3)	0.048 (3)	0.053 (3)	0.076 (3)
C28B_b	0.077 (3)	0.102 (3)	0.090 (3)	0.034 (2)	0.049 (2)	0.027 (2)
C29B_b	0.077 (3)	0.102 (3)	0.090 (3)	0.034 (2)	0.049 (2)	0.027 (2)
C30B_b	0.077 (3)	0.102 (3)	0.090 (3)	0.034 (2)	0.049 (2)	0.027 (2)
C31B_b	0.077 (3)	0.102 (3)	0.090 (3)	0.034 (2)	0.049 (2)	0.027 (2)
C32B_b	0.077 (3)	0.102 (3)	0.090 (3)	0.034 (2)	0.049 (2)	0.027 (2)
C33B_b	0.077 (3)	0.102 (3)	0.090 (3)	0.034 (2)	0.049 (2)	0.027 (2)
N1	0.0319 (10)	0.0332 (10)	0.0311 (9)	0.0044 (8)	0.0137 (8)	0.0078 (8)
O1	0.0595 (11)	0.0605 (11)	0.0455 (10)	−0.0196 (9)	0.0217 (9)	0.0091 (9)
O2	0.129 (2)	0.0555 (14)	0.147 (3)	−0.0043 (15)	0.094 (2)	−0.0106 (15)
O3	0.0770 (14)	0.0816 (14)	0.0477 (11)	0.0355 (11)	0.0306 (10)	0.0313 (10)
O4	0.0666 (12)	0.0495 (11)	0.0456 (11)	−0.0122 (9)	0.0328 (10)	−0.0060 (8)
Cl1	0.1282 (9)	0.0771 (6)	0.1453 (10)	−0.0597 (6)	0.0716 (8)	−0.0339 (6)
Br1	0.0877 (3)	0.0640 (2)	0.05536 (19)	0.00543 (16)	0.03890 (17)	−0.01486 (14)

### Geometric parameters (Å, º)

**Table:**

C1—C6	1.370 (3)	C18—H18	0.9300
C1—N1	1.380 (3)	C19—H19	0.9300
C1—C2	1.500 (3)	C20—C25	1.381 (3)
C2—C3B_b	1.515 (10)	C20—C21	1.397 (3)
C2—C3A_a	1.525 (4)	C21—O4	1.352 (3)
C2—H2A	0.925 (16)	C21—C22	1.388 (3)
C2—H2B	0.948 (15)	C22—C23	1.378 (4)
C3A_a—C4A_a	1.485 (4)	C22—Br1	1.891 (2)
C3A_a—C14	1.551 (3)	C23—C24	1.370 (4)
C3A_a—H3A_a	0.9800	C23—H23	0.9300
C4A_a—C5	1.490 (4)	C24—C25	1.381 (4)
C4A_a—H4A1_a	0.9700	C24—Cl1	1.744 (3)
C4A_a—H4A2_a	0.9700	C25—H25	0.9300
C3B_b—C14	1.684 (11)	C26—N1	1.476 (3)
C3B_b—H3B_b	0.9800	C26—C27	1.488 (4)
C5—O1	1.264 (3)	C26—H26A	0.9700
C5—C6	1.424 (3)	C26—H26B	0.9700
C6—C7	1.510 (3)	C27—O2	1.402 (4)
C7—C8	1.494 (3)	C27—H27A	0.9700
C7—C20	1.544 (3)	C27—H27B	0.9700
C7—H7	0.9800	C28A_a—C29A_a	1.3900
C8—C9	1.356 (3)	C28A_a—C33A_a	1.3900
C8—C13	1.464 (3)	C29A_a—C30A_a	1.3900
C9—N1	1.403 (3)	C29A_a—H29A_a	0.9300
C9—C10	1.501 (3)	C30A_a—C31A_a	1.3900
C10—C11A_a	1.497 (10)	C30A_a—H30A_a	0.9300
C10—C11B_b	1.544 (10)	C31A_a—C32A_a	1.3900
C10—H10A	0.968 (17)	C31A_a—H31A_a	0.9300
C10—H10B	0.964 (18)	C32A_a—C33A_a	1.3900
C11A_a—C12	1.388 (11)	C32A_a—H32A_a	0.9300
C11A_a—C28A_a	1.452 (12)	C33A_a—H33A_a	0.9300
C11B_b—C12	1.303 (11)	C28B_b—C29B_b	1.3900
C11B_b—C28B_b	1.690 (12)	C28B_b—C33B_b	1.3900
C12—C13	1.502 (4)	C29B_b—C30B_b	1.3900
C12—H12	0.889 (18)	C29B_b—H29B_b	0.9300
C13—O3	1.216 (3)	C30B_b—C31B_b	1.3900
C14—C15	1.3900	C30B_b—H30B_b	0.9300
C14—C19	1.3900	C31B_b—C32B_b	1.3900
C15—C16	1.3900	C31B_b—H31B_b	0.9300
C15—H15	0.9300	C32B_b—C33B_b	1.3900
C16—C17	1.3900	C32B_b—H32B_b	0.9300
C16—H16	0.9300	C33B_b—H33B_b	0.9300
C17—C18	1.3900	O2—H2O	0.873 (19)
C17—H17	0.9300	O4—H4O	0.826 (17)
C18—C19	1.3900		
			
C6—C1—N1	119.67 (19)	C18—C17—H17	120.0
C6—C1—C2	122.09 (19)	C16—C17—H17	120.0
N1—C1—C2	118.19 (18)	C17—C18—C19	120.0
C1—C2—C3B_b	109.6 (4)	C17—C18—H18	120.0
C1—C2—C3A_a	112.4 (2)	C19—C18—H18	120.0
C1—C2—H2A	110.5 (15)	C18—C19—C14	120.0
C3B_b—C2—H2A	98.2 (10)	C18—C19—H19	120.0
C3A_a—C2—H2A	123.8 (11)	C14—C19—H19	120.0
C1—C2—H2B	111.0 (15)	C25—C20—C21	118.8 (2)
C3B_b—C2—H2B	130.1 (15)	C25—C20—C7	120.6 (2)
C3A_a—C2—H2B	103.0 (15)	C21—C20—C7	120.6 (2)
H2A—C2—H2B	93.6 (19)	O4—C21—C22	117.4 (2)
C4A_a—C3A_a—C2	111.7 (2)	O4—C21—C20	123.0 (2)
C4A_a—C3A_a—C14	112.8 (2)	C22—C21—C20	119.5 (2)
C2—C3A_a—C14	108.1 (2)	C23—C22—C21	121.1 (2)
C4A_a—C3A_a—H3A_a	108.0	C23—C22—Br1	119.11 (19)
C2—C3A_a—H3A_a	108.0	C21—C22—Br1	119.73 (19)
C14—C3A_a—H3A_a	108.0	C24—C23—C22	119.0 (2)
C3A_a—C4A_a—C5	109.5 (2)	C24—C23—H23	120.5
C3A_a—C4A_a—H4A1_a	109.8	C22—C23—H23	120.5
C5—C4A_a—H4A1_a	109.8	C23—C24—C25	120.9 (3)
C3A_a—C4A_a—H4A2_a	109.8	C23—C24—Cl1	119.8 (2)
C5—C4A_a—H4A2_a	109.8	C25—C24—Cl1	119.3 (2)
H4A1_a—C4A_a—H4A2_a	108.2	C20—C25—C24	120.7 (2)
C2—C3B_b—C14	102.1 (6)	C20—C25—H25	119.6
C2—C3B_b—H3B_b	105.1	C24—C25—H25	119.6
C14—C3B_b—H3B_b	105.1	N1—C26—C27	111.4 (2)
O1—C5—C6	121.4 (2)	N1—C26—H26A	109.3
O1—C5—C4A_a	118.8 (2)	C27—C26—H26A	109.3
C6—C5—C4A_a	119.8 (2)	N1—C26—H26B	109.3
C1—C6—C5	119.4 (2)	C27—C26—H26B	109.3
C1—C6—C7	120.38 (19)	H26A—C26—H26B	108.0
C5—C6—C7	120.09 (18)	O2—C27—C26	107.7 (2)
C8—C7—C6	108.03 (17)	O2—C27—H27A	110.2
C8—C7—C20	112.89 (18)	C26—C27—H27A	110.2
C6—C7—C20	111.46 (17)	O2—C27—H27B	110.2
C8—C7—H7	108.1	C26—C27—H27B	110.2
C6—C7—H7	108.1	H27A—C27—H27B	108.5
C20—C7—H7	108.1	C29A_a—C28A_a—C33A_a	120.0
C9—C8—C13	120.7 (2)	C29A_a—C28A_a—C11A_a	134.8 (10)
C9—C8—C7	121.09 (19)	C33A_a—C28A_a—C11A_a	104.4 (10)
C13—C8—C7	118.21 (19)	C30A_a—C29A_a—C28A_a	120.0
C8—C9—N1	120.02 (19)	C30A_a—C29A_a—H29A_a	120.0
C8—C9—C10	122.7 (2)	C28A_a—C29A_a—H29A_a	120.0
N1—C9—C10	117.18 (18)	C31A_a—C30A_a—C29A_a	120.0
C11A_a—C10—C9	116.2 (4)	C31A_a—C30A_a—H30A_a	120.0
C9—C10—C11B_b	110.0 (4)	C29A_a—C30A_a—H30A_a	120.0
C11A_a—C10—H10A	92.7 (18)	C30A_a—C31A_a—C32A_a	120.0
C9—C10—H10A	111.5 (17)	C30A_a—C31A_a—H31A_a	120.0
C11B_b—C10—H10A	117.0 (17)	C32A_a—C31A_a—H31A_a	120.0
C11A_a—C10—H10B	116 (2)	C33A_a—C32A_a—C31A_a	120.0
C9—C10—H10B	109 (2)	C33A_a—C32A_a—H32A_a	120.0
C11B_b—C10—H10B	98 (2)	C31A_a—C32A_a—H32A_a	120.0
H10A—C10—H10B	111 (3)	C32A_a—C33A_a—C28A_a	120.0
C12—C11A_a—C28A_a	119.6 (10)	C32A_a—C33A_a—H33A_a	120.0
C12—C11A_a—C10	117.6 (8)	C28A_a—C33A_a—H33A_a	120.0
C28A_a—C11A_a—C10	110.0 (8)	C29B_b—C28B_b—C33B_b	120.0
C12—C11B_b—C10	120.0 (9)	C29B_b—C28B_b—C11B_b	108.9 (7)
C12—C11B_b—C28B_b	114.4 (9)	C33B_b—C28B_b—C11B_b	130.8 (7)
C10—C11B_b—C28B_b	101.5 (7)	C28B_b—C29B_b—C30B_b	120.0
C11B_b—C12—C13	116.5 (5)	C28B_b—C29B_b—H29B_b	120.0
C11A_a—C12—C13	122.0 (4)	C30B_b—C29B_b—H29B_b	120.0
C11B_b—C12—H12	125 (2)	C31B_b—C30B_b—C29B_b	120.0
C11A_a—C12—H12	125 (2)	C31B_b—C30B_b—H30B_b	120.0
C13—C12—H12	113 (2)	C29B_b—C30B_b—H30B_b	120.0
O3—C13—C8	121.3 (2)	C32B_b—C31B_b—C30B_b	120.0
O3—C13—C12	121.5 (2)	C32B_b—C31B_b—H31B_b	120.0
C8—C13—C12	117.2 (2)	C30B_b—C31B_b—H31B_b	120.0
C15—C14—C19	120.0	C33B_b—C32B_b—C31B_b	120.0
C15—C14—C3A_a	127.33 (19)	C33B_b—C32B_b—H32B_b	120.0
C19—C14—C3A_a	112.64 (19)	C31B_b—C32B_b—H32B_b	120.0
C15—C14—C3B_b	96.4 (5)	C32B_b—C33B_b—C28B_b	120.0
C19—C14—C3B_b	143.5 (5)	C32B_b—C33B_b—H33B_b	120.0
C16—C15—C14	120.0	C28B_b—C33B_b—H33B_b	120.0
C16—C15—H15	120.0	C1—N1—C9	119.12 (17)
C14—C15—H15	120.0	C1—N1—C26	121.61 (17)
C15—C16—C17	120.0	C9—N1—C26	119.15 (17)
C15—C16—H16	120.0	C27—O2—H2O	111.2 (15)
C17—C16—H16	120.0	C21—O4—H4O	116 (2)
C18—C17—C16	120.0		
			
C6—C1—C2—C3B_b	−28.8 (6)	C2—C3B_b—C14—C15	115.5 (6)
N1—C1—C2—C3B_b	148.6 (6)	C2—C3B_b—C14—C19	−59.9 (10)
C6—C1—C2—C3A_a	6.9 (3)	C2—C3B_b—C14—C3A_a	−66.1 (7)
N1—C1—C2—C3A_a	−175.7 (2)	C19—C14—C15—C16	0.0
C1—C2—C3A_a—C4A_a	−43.6 (3)	C3A_a—C14—C15—C16	−177.9 (2)
C3B_b—C2—C3A_a—C4A_a	48.4 (7)	C3B_b—C14—C15—C16	−176.9 (4)
C1—C2—C3A_a—C14	−168.2 (2)	C14—C15—C16—C17	0.0
C3B_b—C2—C3A_a—C14	−76.2 (8)	C15—C16—C17—C18	0.0
C2—C3A_a—C4A_a—C5	57.4 (3)	C16—C17—C18—C19	0.0
C14—C3A_a—C4A_a—C5	179.4 (2)	C17—C18—C19—C14	0.0
C1—C2—C3B_b—C14	161.7 (4)	C15—C14—C19—C18	0.0
C3A_a—C2—C3B_b—C14	60.5 (7)	C3A_a—C14—C19—C18	178.23 (18)
C3A_a—C4A_a—C5—O1	145.7 (3)	C3B_b—C14—C19—C18	174.7 (6)
C3A_a—C4A_a—C5—C6	−37.1 (4)	C8—C7—C20—C25	24.8 (3)
N1—C1—C6—C5	−162.5 (2)	C6—C7—C20—C25	−97.0 (2)
C2—C1—C6—C5	14.9 (3)	C8—C7—C20—C21	−156.9 (2)
N1—C1—C6—C7	13.0 (3)	C6—C7—C20—C21	81.3 (2)
C2—C1—C6—C7	−169.60 (19)	C25—C20—C21—O4	−179.2 (2)
O1—C5—C6—C1	178.0 (2)	C7—C20—C21—O4	2.5 (3)
C4A_a—C5—C6—C1	0.9 (3)	C25—C20—C21—C22	−0.9 (3)
O1—C5—C6—C7	2.5 (3)	C7—C20—C21—C22	−179.2 (2)
C4A_a—C5—C6—C7	−174.6 (2)	O4—C21—C22—C23	−179.9 (2)
C1—C6—C7—C8	−34.4 (3)	C20—C21—C22—C23	1.7 (4)
C5—C6—C7—C8	141.1 (2)	O4—C21—C22—Br1	−1.7 (3)
C1—C6—C7—C20	90.2 (2)	C20—C21—C22—Br1	179.89 (17)
C5—C6—C7—C20	−94.3 (2)	C21—C22—C23—C24	−1.5 (4)
C6—C7—C8—C9	30.6 (3)	Br1—C22—C23—C24	−179.7 (2)
C20—C7—C8—C9	−93.1 (3)	C22—C23—C24—C25	0.4 (5)
C6—C7—C8—C13	−148.5 (2)	C22—C23—C24—Cl1	179.8 (2)
C20—C7—C8—C13	87.8 (2)	C21—C20—C25—C24	−0.2 (4)
C13—C8—C9—N1	173.4 (2)	C7—C20—C25—C24	178.2 (2)
C7—C8—C9—N1	−5.8 (3)	C23—C24—C25—C20	0.4 (4)
C13—C8—C9—C10	−3.4 (4)	Cl1—C24—C25—C20	−179.0 (2)
C7—C8—C9—C10	177.5 (2)	N1—C26—C27—O2	−76.8 (3)
C8—C9—C10—C11A_a	13.4 (9)	C12—C11A_a—C28A_a—C29A_a	−84.2 (16)
N1—C9—C10—C11A_a	−163.4 (9)	C10—C11A_a—C28A_a—C29A_a	56.4 (14)
C8—C9—C10—C11B_b	−13.5 (8)	C12—C11A_a—C28A_a—C33A_a	106.7 (17)
N1—C9—C10—C11B_b	169.7 (7)	C10—C11A_a—C28A_a—C33A_a	−112.6 (9)
C9—C10—C11A_a—C12	−21.4 (17)	C33A_a—C28A_a—C29A_a—C30A_a	0.0
C11B_b—C10—C11A_a—C12	60.2 (15)	C11A_a—C28A_a—C29A_a—C30A_a	−167.7 (9)
C9—C10—C11A_a—C28A_a	−162.9 (10)	C28A_a—C29A_a—C30A_a—C31A_a	0.0
C11B_b—C10—C11A_a—C28A_a	−81 (2)	C29A_a—C30A_a—C31A_a—C32A_a	0.0
C11A_a—C10—C11B_b—C12	−71 (2)	C30A_a—C31A_a—C32A_a—C33A_a	0.0
C9—C10—C11B_b—C12	38.3 (14)	C31A_a—C32A_a—C33A_a—C28A_a	0.0
C11A_a—C10—C11B_b—C28B_b	56.2 (14)	C29A_a—C28A_a—C33A_a—C32A_a	0.0
C9—C10—C11B_b—C28B_b	165.4 (6)	C11A_a—C28A_a—C33A_a—C32A_a	171.0 (6)
C10—C11B_b—C12—C11A_a	65.9 (18)	C12—C11B_b—C28B_b—C29B_b	−143.3 (12)
C28B_b—C11B_b—C12—C11A_a	−54.9 (14)	C10—C11B_b—C28B_b—C29B_b	86.0 (8)
C10—C11B_b—C12—C13	−43.0 (15)	C12—C11B_b—C28B_b—C33B_b	30.3 (17)
C28B_b—C11B_b—C12—C13	−163.8 (7)	C10—C11B_b—C28B_b—C33B_b	−100.3 (7)
C28A_a—C11A_a—C12—C11B_b	71 (2)	C33B_b—C28B_b—C29B_b—C30B_b	0.0
C10—C11A_a—C12—C11B_b	−67.0 (16)	C11B_b—C28B_b—C29B_b—C30B_b	174.5 (6)
C28A_a—C11A_a—C12—C13	158.0 (11)	C28B_b—C29B_b—C30B_b—C31B_b	0.0
C10—C11A_a—C12—C13	20.3 (19)	C29B_b—C30B_b—C31B_b—C32B_b	0.0
C9—C8—C13—O3	−177.3 (3)	C30B_b—C31B_b—C32B_b—C33B_b	0.0
C7—C8—C13—O3	1.9 (4)	C31B_b—C32B_b—C33B_b—C28B_b	0.0
C9—C8—C13—C12	1.0 (4)	C29B_b—C28B_b—C33B_b—C32B_b	0.0
C7—C8—C13—C12	−179.9 (3)	C11B_b—C28B_b—C33B_b—C32B_b	−173.1 (8)
C11B_b—C12—C13—O3	−159.3 (9)	C6—C1—N1—C9	16.0 (3)
C11A_a—C12—C13—O3	168.3 (11)	C2—C1—N1—C9	−161.48 (19)
C11B_b—C12—C13—C8	22.4 (10)	C6—C1—N1—C26	−168.0 (2)
C11A_a—C12—C13—C8	−10.0 (12)	C2—C1—N1—C26	14.6 (3)
C4A_a—C3A_a—C14—C15	−52.8 (3)	C8—C9—N1—C1	−19.9 (3)
C2—C3A_a—C14—C15	71.2 (3)	C10—C9—N1—C1	157.0 (2)
C4A_a—C3A_a—C14—C19	129.2 (2)	C8—C9—N1—C26	163.9 (2)
C2—C3A_a—C14—C19	−106.9 (2)	C10—C9—N1—C26	−19.1 (3)
C4A_a—C3A_a—C14—C3B_b	−54.9 (7)	C27—C26—N1—C1	98.1 (2)
C2—C3A_a—C14—C3B_b	69.1 (7)	C27—C26—N1—C9	−85.8 (2)

### Hydrogen-bond geometry (Å, º)

Cg6, Cg7 and Cg9 are the centroids of the C28B_B–C33B_B, C14–C19 and C28A_A–C33A_A phenyl rings, respectively.

**Table:**

*D*—H···*A*	*D*—H	H···*A*	*D*···*A*	*D*—H···*A*
O2—H2*O*···O1^i^	0.87 (2)	1.93 (2)	2.782 (3)	167 (5)
O4—H4*O*···O1	0.83 (3)	1.84 (3)	2.632 (2)	161 (3)
C10—H10*A*···O3^ii^	0.97 (2)	2.54 (2)	3.211 (3)	126 (2)
C31*B*_*b*—H31*B*_*b*···Cl1^iii^	0.93	2.76	3.530 (7)	141
C26—H26*B*···O3^ii^	0.97	2.57	3.537 (3)	173
C16—H16···*Cg*6^iv^	0.93	2.89	3.713 (4)	149
C16—H16···*Cg*9^iv^	0.93	2.86	3.718 (4)	154
C27—H27*B*···*Cg*7^v^	0.97	2.71	3.574 (3)	149

Symmetry codes: (i) −*x*, *y*+1/2, −*z*+1/2; (ii) *x*, −*y*+1/2, *z*−1/2; (iii) −*x*+1, *y*−1/2, −*z*+1/2; (iv) *x*−1, −*y*+1/2, *z*−1/2; (v) −*x*, −*y*+1, −*z*.
